# Prognostic value of Kindlin-2 expression in patients with solid tumors: a meta-analysis

**DOI:** 10.1186/s12935-018-0651-7

**Published:** 2018-10-22

**Authors:** Sheng Liu, Sheng Chen, Kaige Ma, Zengwu Shao

**Affiliations:** Department of Orthopaedics, Union Hospital, Tongji Medical College, Huazhong University of Science and Technology, Wuhan, 430022 China

**Keywords:** Kindlin-2, Solid tumor, Cancer, Prognosis, Meta-analysis

## Abstract

**Background:**

Kindlin-2 is one of the Kindlin family members which are evolutionarily conserved focal adhesion proteins with integrin β-binding affinity. Recently, accumulative studies have suggested that Kindlin-2 plays important roles in tumor biology. However, the prognostic significance of Kindlin-2 in patients with solid tumors remains controversial. Therefore, this study aimed to clarify the prognostic value of Kindlin-2 in solid tumors via meta-analysis.

**Methods:**

A comprehensive search was performed in PubMed, Embase, Web of Science and EBSCO for all relevant studies reporting the prognostic significance of Kindlin-2 expression in solid cancer patients. The summary hazard ratio (HR) and corresponding 95% confidence interval (CI) were calculated to estimate the association between Kindlin-2 expression with survival of solid cancer patients.

**Results:**

We included 14 eligible studies containing 1869 patients in our meta-analysis. The pooled results indicated that high Kindlin-2 expression was significantly associated with poor overall survival (OS) (pooled HR 1.66, 95% CI 1.44–1.92, *P *< 0.0001), disease-free survival (DFS)/recurrence-free survival (RFS)/progression-free survival (PFS) (pooled HR 1.73, 95% CI 1.16–2.57, *P *= 0.0067). For certain tumor types, high Kindlin-2 expression was significantly correlated with a poor outcome in patients with solid tumors, including pancreatic ductal adenocarcinoma (DFS/RFS/PFS), esophageal squamous cell carcinoma (OS, DFS/RFS/PFS), hepatocellular carcinoma (OS), clear cell renal cell carcinoma (OS), bladder cancer (OS, DFS/RFS/PFS), chondrosarcoma (OS), osteosarcoma (OS), gastric cancer (DFS/RFS/PFS), and glioma (OS).

**Conclusions:**

Our meta-analysis demonstrated that high Kindlin-2 expression might indicate poor outcome in patients with solid tumors and could serve as a prognostic biomarker for solid cancer patients.

## Background

Cancer is one of the leading contributors to heavy health care burden and disease-related mortality worldwide, with approximately 1,735,350 new cancer cases and 609,640 cancer-related deaths in the United States in 2018 [1, 2]. Although great advances in early detection and treatments have been made in recent years, the prognosis of cancer patients is still poor [3, 4]. Therefore, novel prognostic biomarkers are urgently needed for precisely predicting the outcome and providing therapeutic targets for cancer patients.

The Kindlin family is composed of three members of evolutionarily conserved focal adhesion proteins (Kindlin-1, -2 and -3) in mammal, which share the same 4.1-ezrin-radixin-moesin (FERM) domain, but have different expression distribution [5]. Kindlins can exert extensive biological functions in cell proliferation, migration, differentiation and cell death through binding with integrin β cytoplasmic tails and activating integrins, which have been linked to many hereditary disease and acquired disease of human [6]. Kindlin-1 (also known as FERMT1) is highly expressed in the skin and other tissues, whose deficiency and mutation can cause Kindler Syndrome [7, 8]. Kindlin-3 (also known as FERMT3) is generally expressed in the notochord, central nervous system, cement gland, and etc., mutations in which can contribute to leukocyte adhesion deficiency type III [8, 9].

Kindlin-2 (also known as FERMT2) was detected in various cell types, including fibroblast cells, smooth muscle cells and endothelial cells [10]. As a broadly distributed focal adhesion protein, Kindlin-2 has binding sites for various interaction partners, such as integrin, actin, the filamin-binding protein migfilin, integrin-linked kinase (ILK) [11, 12]. Previous studies demonstrated that Kindlin-2 could interact with integrin and these partners to activate Wnt signaling, transforming growth factor β (TGF-β) signaling,epidermal growth factor receptor (EGFR) signaling, Hedgehog and extracellular regulated protein kinases (ERK) signaling pathways, which play vital roles in tumor progression [13]. Recently, increasing evidences indicated the correlation between Kindlin-2 expression and prognosis in various types of solid tumors [14–28]. However, several studies demonstrated negative role or no significant association [14, 24, 28, 29]. Therefore, we performed this meta-analysis to explore the prognostic value of Kindlin-2 expression in patients with solid tumors.

## Materials and methods

### Study strategy

This meta-analysis study was based on the Preferred Reporting Items for Systematic Reviews and Meta-Analyses (PRISMA) guidelines [30]. Two authors (Sheng Liu and Sheng Chen) independently carried out the search. PubMed, Embase, Web of Science and EBSCO were searched for articles reporting the prognostic role of Kindlin-2 expression in patients with solid tumors. The search strategy based on MeSH words was “Kindlin-2 OR FEMRT2 OR pleckstrin homology domain-containing family C member 1 (PLEKHC1) OR uncoordinated protein 112 (UNC112) OR mitogen-inducible gene-2 (MIG-2) OR UNC112 related protein 2 short form (URP2SF)” AND “tumor OR neoplasm OR cancer OR carcinoma OR malignancy” AND “prognosis OR prognostic OR survival”. The retrieval ended on 10 July, 2018. The references lists in identified articles were screened carefully lest relevant studies should be omitted.

### Inclusion and exclusion criteria

We included all articles meeting the criteria as follows: (1) cohort study; (2) Kindlin-2 expression in cancer tissue or relevant tissue; (3) the prognostic outcome of Kindlin-2 different expression group; (4) available data such as Kaplan–Meier (KM) plot, the hazard ratio (HR) and 95% confidence intervals (CI). Studies of non-human research, reviews, letters, case reports, laboratory articles, non-English articles and conference abstracts were excluded. Two authors (Sheng Liu and Sheng Chen) independently screened the titles and abstracts of identified articles, and excluded those considered irrelevant. Further evaluation was conducted by viewing the full text carefully. Disagreements were resolved by consulting with a third author (Zengwu Shao).

### Data extraction

Two researchers (Sheng Liu and Sheng Chen) independently extracted the relevant data from all eligible articles. The following data of each study was extracted: first author, publication year, original country, number of enrolled patients, tumor type, detected methods, cut-off value, high expression presentations, follow-up time, and HR and 95% CI of the high Kindlin-2 expression group versus the low one for various outcomes. The HR and 95% CI were extracted preferentially from multivariable analyses such as Cox proportional-hazards model. When the HRs were not provided, we extracted the survival information from the original study data (KM plot or the required data) using the software Engauge Digitizer 10.5 [31] and estimated the survival data by Tierney’s method [32].

### Quality assessment

The quality of each study was assessed by two investigators (Sheng Liu and Sheng Chen) independently using the Newcastle–Ottawa Quality Assessment Scale (NOS). Any disagreement was resolved by discussing with another investigator (Kaige Ma). The scales allocate the total score for each study ranged from 0 to 9 for the quality of selection, comparability, exposure and outcomes of included studies. The studies with scores ≥ 6 were considered as high-quality studies.

### Statistical analysis

The statistical analysis was performed using the software R 3.4.4 [33], meta package [34] and meta for package [35]. Pooled HRs and their corresponding 95% CIs were used to describe the prognostic value of Kindlin-2 expression. The heterogeneity was assessed using the Cochran Q-test and I-squared test. If I^2^ < 50% or *P* > 0.05, it was indicated that no heterogeneity existed among studies, and a fixed-effects model was performed. Otherwise, it was considered as significant heterogeneity and the random-effects model was applied. Meta-regression and subgroup analysis were performed with the studies sorted into subgroups according to similar variables. Sensitivity analysis was applied to evaluate the stability of the results. Funnel plot and Egger’s test were applied to assess the potential publication bias. Statistical significance was defined as *P* value < 0.05.

## Results

### Eligible studies and their characteristics

**Fig. 1:**
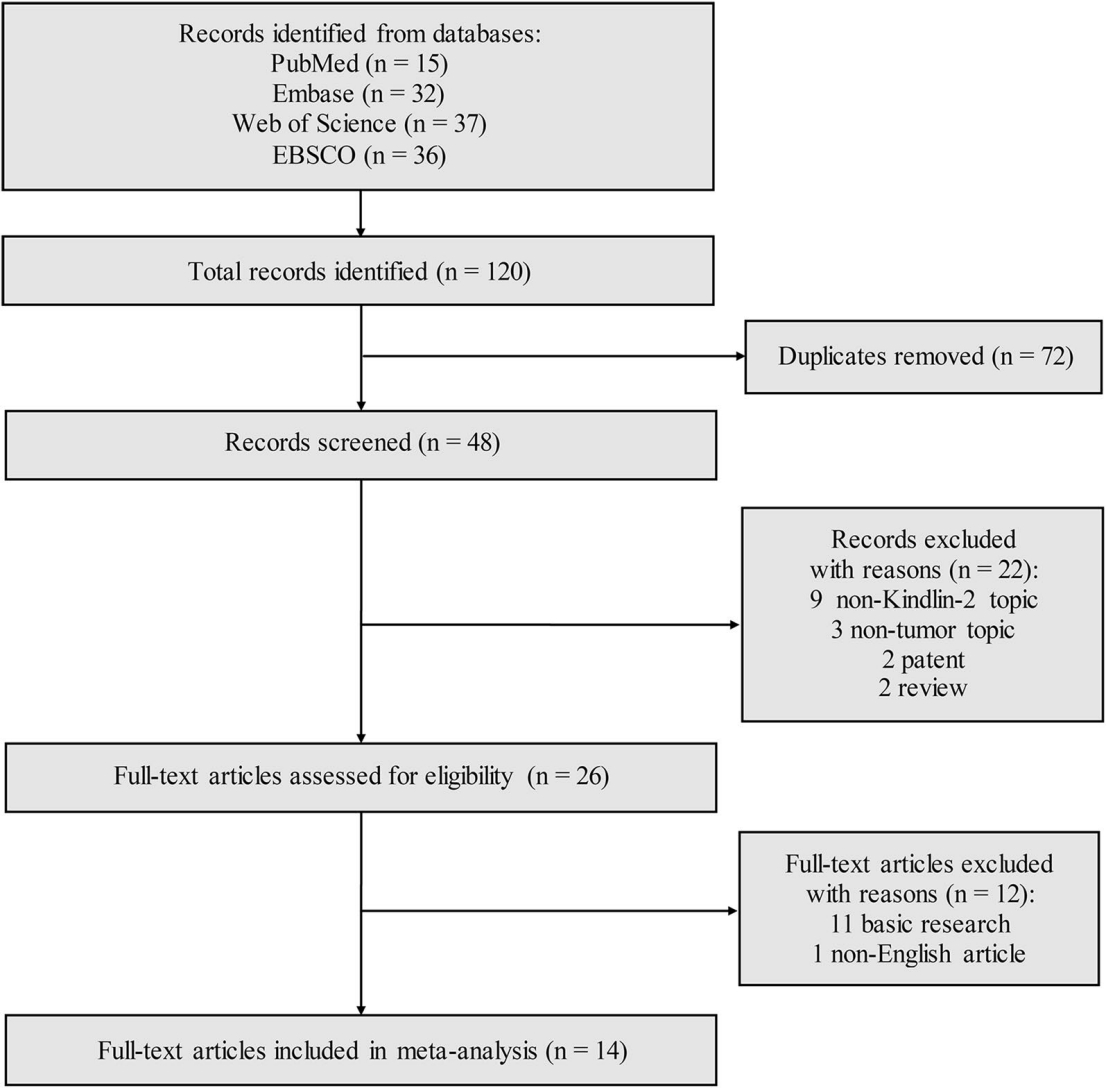
Flow diagram of the study selection process

According to the searching strategy above mentioned, 120 records were retrieved from the databases. After 72 duplicated records were removed, the remaining articles were screened. Then, 22 of 48 records were excluded because of several reasons: nine articles did not report Kindlin-2 expression as a prognostic variable; three did not involve a tumor; the remaining articles were six meeting articles, two patent articles and two review articles. When the further full-text review was finished, eleven basic research articles and one in non-English were excluded. Finally, the meta-analysis was performed for the remaining 14 articles (Fig. 1).

The included articles all had cohort study and published in the recent decade (2008–2017). In total, 1869 patients in the 16 cohorts were enrolled from China, Japan and Greece. They were diagnosed with pancreatic ductal adenocarcinoma (PDAC), esophageal squamous cell carcinoma (ESCC), bladder cancer (BC), chondrosarcoma (CHS), hepatocellular carcinoma (HCC), osteosarcoma (OSS), glioma, serous epithelial ovarian cancers (sEOC), gastric cancer (GC), or clear cell renal cell carcinoma (ccRCC). The expression of Kindlin-2 was detected by immunohistochemistry (IHC) or Western Blot (WB) in these studies, although the cut-off value varied in different studies. At least overall survival (OS) was used as the prognostic outcome in every study. HRs with their 95% CIs based on Cox proportional-hazards model (Cox) were reported in 11 studies directly. In the remaining three studies, the data were calculated from the KM plots or the *P*-value of log-rank test. Every study’s NOS score was more than 6 points, which meant favorable methodology. The main characteristics of the eligible studies were summarized in Table 1. And the main clinicopathologic features and their distribution of patients in these studies were shown on Table 2. Kindlin-2 expression was reported to have a significant association with several variables, including age, tumor size, stage, tumor category, lymphatic and vascular invasion, metastasis and response to chemotherapy (*P *< 0.05) (Table 2).

**Table 1:** The main characteristics of the eligible studies

Study (Author and year)	Country	size	Tumor type	Sample type	Method (antibody data)	Negative control	Expression location	Cut-off value: (intention) or (IPS = x * y)	High expression ratio: n/N (%)	Follow-up time: mean (min–max) (mon)	Survival outcome	Conclusion (UA/MA)	Multivariate analysis	HR extraction	NOS score
Yoshida et al. 2017 (I) [14]	Japan	79	PDAC	ac (Ca) Startle (St)	IHC (M, Merck Millipore)	NT + NP	NR	50%	Ca: 54/79 (68%) St: 49/79 (62%)	NR	OS; RFS OS; RFS	NS; NS; NS; P	No	KM plot	8
Zhan et al. 2015 [15]	China	31	PDAC	ac	IHC (Millipore)	PBS	NR	50%	15/31 (48%)	47 (3–73)	OS	P	No	P-value	7
Mahawithitwong et al. 2013 [16]	Japan	95	PDAC	sf	IHC (R, Protein TechGroup, 1:100)	NR	NR	(4 * 3) 4/12	34/95 (64.2%)	24 (3–136); 14 (0–136)	OS; DFS	P/NS; P/−	Yes	Cox, P-value	7
Cao et al. 2015 (II) [17]	China	110	ESCC	scc	IHC (M, Origen,1:50)	NR	C + N	(3 * 4) NR	34/65 (52%)	36.5 (0–148.7)	OS; DFS	−/P; −/P	Yes	Cox	6
Cao et al. 2015 (III) [17]	China	147	ESCC	scc	IHC (M, Origen,1:50)	NR	C + N	(3 * 4) NR	20/64 (31%)	28.8 (27–72)	OS; DFS	P; P	Yes	Cox	6
Wu et al. 2017 [18]	China	203	BC	sf	IHC (M, Santa Cruz, 1:500)	PBS	NR	(3 * 4) 6/12	109/203 (54%)	64 (49–78)	OS; CSS; DFS	P/P; P/−; P/−	Yes	Cox	6
Papachristou et al. 2008 [19]	Greece etc.	60	CHS	sf	IHC (M, homemade, 1/50)	TBS	C	33%	51/60 (85%)	67.9 (40.9, 2–180)	OS	P	No	P-value	8
Ge et al. 2015 [20]	China	72	HCC	ac	IHC (R, ab152106, 1:100)	PBS	NR	(4 * 3) 4/12	43/72 (60%)	NR (17.96–43.11)	OS; DFS	P/P; P/P	Yes	Cox	7
Lin et al. 2017 [21]	China	127	HCC	ac	IHC (M, MAB2617, Billerica, 1:100)	NR	C	(3 * 4) 4/12	103/127 (81%)	22 (1–94)	OS	P/P	Yes	Cox	8
Ning et al. 2017 [22]	China	100	OSS	Sarcoma	IHC (R, Millipore, 1:150)	PBS	N	(3 * 4) 4.56/12	51/100 (51%)	29.82 (5.26–38.89)	OS; DFS	P/P; P/P	Yes	Cox	7
Ou et al. 2016 [23]	China	188	Glioma	Carcinoma	IHC (1:100)	NR	NR	4/12	132/188 (70%)	NR (0–39)	OS	P/P	Yes	Cox	8
Ren et al. 2014 [24]	China	113	sEOC	scc	IHC (R, Dako, 1:2000)	PBS	NR	(4 * 4) 12/16	91/113 (80%)	NR	OS; PFS	N/NS; N/N	Yes	KM-plot	6
Shen et al. 2012 [25]	China	40	GC	ac	WB (R, ab74030, Abcam, 1:600)	actin	NR	Ratio: K2/actin > 2	22/40 (55%)	37.1 (5–77)	OS; PFS	P/NS; P/P	Yes	Cox	8
Li et al. 2017 [26]	China	109	ccRCC	ac	IHC (M, Millipore)	NT	C	50%	70/109 (64%)	69 (0.94–82)	OS	P/NS	Yes	Cox	7
Yan et al. 2016 [27]	China	336	ccRCC	ac	IHC (M, ab117962, Abcam, 1:100)	NR	NR	(3 * 3) 4/9	199/336 (59%)	NR (10–60)	OS; DFS	−/P; −/P	Yes	Cox	7

(I) This article (Yoshida [14]) was listed two cohort study because the sample types contains cancer tissue and startle cell. (II) and (III) This article (Cao [17]) included patients from generation dataset (II) and validation dataset (III). Antibody data mainly contains the species (mouse, rabbit), code, manufacturer, and concentration ratio

n: number of patients; PDAC: pancreatic ductal adenocarcinoma; ESCC: esophageal squamous cell carcinoma; BC: bladder cancer; CHS: chondrosarcoma; HCC: hepatocellular carcinoma; OSS: osteosarcoma; sEOC: serous epithelial ovarian cancers; GC: gastric cancer; ccRCC: clear cell renal cell carcinoma; ac: adenocarcinoma; Ca: cancer tissue, St: startle cell; sf: stromal fibroblasts; scc: squamous cell carcinoma; IHC: immunohistochemistry; WB: Western Blot; NR: no report; NT: non-cancer tissue; NP: non-tumor patient; PBS: phosphate buffered solution; TBS: triethanolamine buffered solution; C: cytoplasm, N: cellular nucleus; IPS: immunohistochemical positive score; x: up-limit of the averaged staining intensity score; y: up-limit of the score standing for stained cells proportion; *: multiplication of the two score; Ratio: the ratio of gray value; UA: univariate analysis; MA: multivariate analysis; NS: not significant, P: positive for the conclusion that Kindlin-2 high expression is associated with poor prognostic outcome, N: negative for the conclusion; Cox: Cox proportional-hazards model; NOS: the Newcastle–Ottawa Quality Assessment Scale

**Table 2:** The main clinicopathologic features of patients and their distribution in the eligible studies

Study (Author and year)	n	Age (years or numbers): [mean or median (range)] (cut-off: low/high)	Sex (M/F)	Histological differentiation (I/II/III)	Tumor size (cm) (cut-off) (low/high)	Tumor category (grade)	Lymphatic invasion (∓)	Vascular invasion (low/high)	Metastasis (∓)	Staging method	Stage (cut-off)	Other therapy (no/yes)
Yoshida et al. 2017 (I) [14]	79	65 (mean) (41–85) (65):39/40	51/28	9/63/7	NR	NR	19/60	32/47	NR	NR	NR	C: 9/70 R: 68/11
Zhan et al. 2015 [15]	31	NR	NR	NR	NR	NR	NR	NR	NR	NR	NR	NR
Mahawithitwong et al. 2013 [16]	95	65 (mean) (36–86) (65): 52/43	58/37	10/33/52	NR	(T1/2/3/4) 9/3/82/1	34/61*	38/57	NR	UICC	NR	C: 10/85 R: 78/17
Cao et al. 2015 (II) [17]	110	(58): 55/55	80/30	33/67/10	(3, 5) 32/45/11	(T1, 2/3, 4) 7/103	57/53	NR	NR	TNM	(IIB/IIIA) 59/51	99/12
Cao et al. 2015 (III)[17]	147	(58): 79/68	113/34	23/109/15	(3, 5) 38/71/36	(T1, 2/3, 4) 20/127	64/83	NR	NR	TNM	(IIB/IIIA) 70/77	104/43
Wu et al. 2017 [18]	203	(65): 109/94	165/38	(Low/high) 96/107*	(3) 140/63	NR	NR	NR	NR	TNM	(I/II) 8/115*	NR
Papachristou et al. 2008 [19]	60	54 (mean) (21–85)	34/26	20/29/11*	(8) 23/37	NR	NR	NR	NR	NR	NR	NR
Ge et al. 2015 [20]	72	(53): 35/37	60/12	NR	(5) 29/43*	NR	NR	Cap: 44/28* Mic: 49/23*	NR	TNM	(II/III) 41/31	NR
Lin et al. 2017 [21]	127	(60): 111/16	17/110	NR	(3) 10/117	NR	NR	Cap: 40/87 Mic: 66/61*	9/115*	NR	(II/III) 11/116	NR
Ning et al. 2017 [22]	100	(18): 40/60	68/32	(Low/high) 15/85*	NR	NR	NR	NR	60/40*	NR	NR	RC: 50/50*
Ou et al. 2016 [23]	188	39 (mean) (39): 98/90*	103/85	NR	NR	NR	NR	NR	NR	NR	(II/III) 85/103*	NR
Ren et al. 2014 [24]	113	(50): 28/85*	−/113	(Low/high) 26/87*	NR	NR	NR	NR	49/34	FIGO	(I/II/III/IV) 9/13/73/10	RC: 21/68
Shen et al. 2012 [25]	40	67 (mean) (47–93) (60): 14/26	30/10	4/8/28	NR	(T1, 2/3, 4) 8/32*	N1/2/3 21/10/9*	NR	37/3	Pathologic	(II/III) 8/32*	NR
Li et al. 2017 [26]	109	(60):62/47	67/42	36/41/32*	NR	(Tx/1/2/34) 4/68/20/17	Nx/0/1 2/99/8*	NR	NR	AJCC	(II/III) 70/39	NR
Yan et al. 2016 [27]	336	(65):177/159	240/96	NR	(4) 176/160	(T1, 2/3, 4) 167/169	202/134	NR	269/67*	TNM	(II/III) 124/212*	NR

(I) This article (Yoshida [14]) was listed two cohort study because the sample types contains cancer tissue and startle cell. (II) and (III) This article (Cao [17]) included patients from generation dataset (II) and validation dataset (III)

n: number of patients; NR: no report; Cap: capillary invasion; Mic: microvascular invasion; C: chemotherapy; R: radiotherapy; RC: response for chemotherapy

*Means that Kindlin-2 expression was reported to have a significant relation with the variable in the study

### Correlation between Kindlin-2 expression and survival outcomes of solid tumors

According to the protocol described above, the meta-analysis was performed and its main results were listed in Table 3. There were four survival outcomes evaluated in the included studies, including OS, disease-free survival (DFS), recurrence-free survival (RFS), progression-free survival (PFS). Given that they are similar in definition and number of studies evaluating RFS and PFS was limited (Table 1), we combined the latter three ones together as DFS/RFS/PFS. Thus, this meta-analysis was conducted with two groups: OS and DFS/RFS/PFS.

**Table 3:** The pooled HR and 95% CI for the prognostic value of Kindlin-2 expression

Outcome group	Subgroup	No. of studies	No. of patients	Model	Pooled HR (95% CI)	P value of pooled HR	Heterogeneity		P value of meta-regression
							I^2^ (%)	P value	
Overall									
OS	Overall	16	1869	Fixed	1.6612 [1.4400; 1.9164]	< 0.0001	36.3	0.0729	–
DFS/RFS/PFS		11	1374	Random	1.7309 [1.1643; 2.5733]	0.0067	76.9	< 0.0001	
Sample size									
OS	≥ 100	9	1433	Random	1.6074 [1.2435; 2.0777]	0.0003	52.5	0.03	0.3455
	< 100	7	436	Fixed	1.9081 [1.3873; 2.6245]	0.0001	0.0	0.45	
DFS/RFS/PFS	≥ 100	6	1009	Random	1.3943 [0.8759; 2.2194]	0.1611	70.7	< 0.01	0.2277
	< 100	5	365	Random	2.2280 [1.1574; 4.2886]	0.0165	78.0	< 0.01	
Tumor type (from which system)									
OS	Digestive	9	780	Fixed	1.7955 [1.4224; 2.2664]	< 0.0001	0.0	0.79	0.5000
	Non-digestive	7	1089	Random	1.6305 [1.1236; 2.3662]	0.0101	67.0	< 0.01	
DFS/RFS/PFS	Digest	7	622	Random	2.0137 [1.2856; 3.1542]	0.0022	72.2	< 0.01	0.3149
	Non-digestive	4	752	Random	1.3101 [0.5547; 3.0945]	0.5378	81.9	< 0.01	
Sample type (from which tissue)									
OS	Cancer	13	1492	Random	1.7897 [1.3855; 2.3118]	< 0.0001	46.1	0.03	0.5741
	Stroma	3	377	Fixed	1.5830 [1.1958; 2.0957]	0.0013	0.0	0.57	
DFS/RFS/PFS	Cancer	8	997	Random	1.8358 [1.0668; 3.1589]	0.0283	83.4	< 0.01	0.6650
	Stroma	3	377	Fixed	1.5566 [1.0726; 2.2590]	0.0199	0.0	0.74	
Max follow-up time (months)									
OS	≥ 60	13	1509	Random	1.6442 [1.3212; 2.0462]	0.0207	31.7	0.13	0.4370
	< 60	3	360	Random	2.4020 [1.1431; 5.0471]	< 0.0001	66.3	0.05	
DFS/RFS/PFS	≥ 60	9	1202	Random	1.4740 [0.9864; 2.2028]	0.0583	76.7	< 0.01	0.0258**
	< 60	2	172	Fixed	4.9891 [2.4072; 10.3405]	< 0.0001	0.0	0.53	
HR extraction									
OS	COX	11	1527	Fixed	1.7024 [1.4600; 1.9851]	< 0.0001	0.0	0.61	0.4737
	Non-COX	5	342	Random	1.6093 [0.7542; 3.4340]	0.2185*	72.7	< 0.01	
DFS/RFS/PFS	COX	8	1103	Random	2.2266 [1.5122; 3.2785]	< 0.0001	72.1	< 0.01	0.0085**
	Non-COX	3	271	Random	0.7158 [0.2982; 1.7182]	0.4542*	66.7	0.05	
NOS score									
OS	≥ 8	6	553	Fixed	1.6820 [1.3178; 2.1470]	< 0.0001	0.0	0.64	0.6371
	< 8	10	1316	Random	1.6701 [1.2539; 2.2243]	0.0005	55.3	0.02	
DFS/RFS/PFS	≥ 8	3	198	Random	1.9211 [0.6133; 6.0179]	0.2624*	86.8	< 0.01	0.6479
	< 8	8	1176	Random	1.6244 [1.0899; 2.4211]	0.0172	69.8	< 0.01	

*Means that the P value of pooled HR is more than 0.05

**Means the P value from the test of moderators in the meta-regression is lower than 0.05

**Fig. 2:**
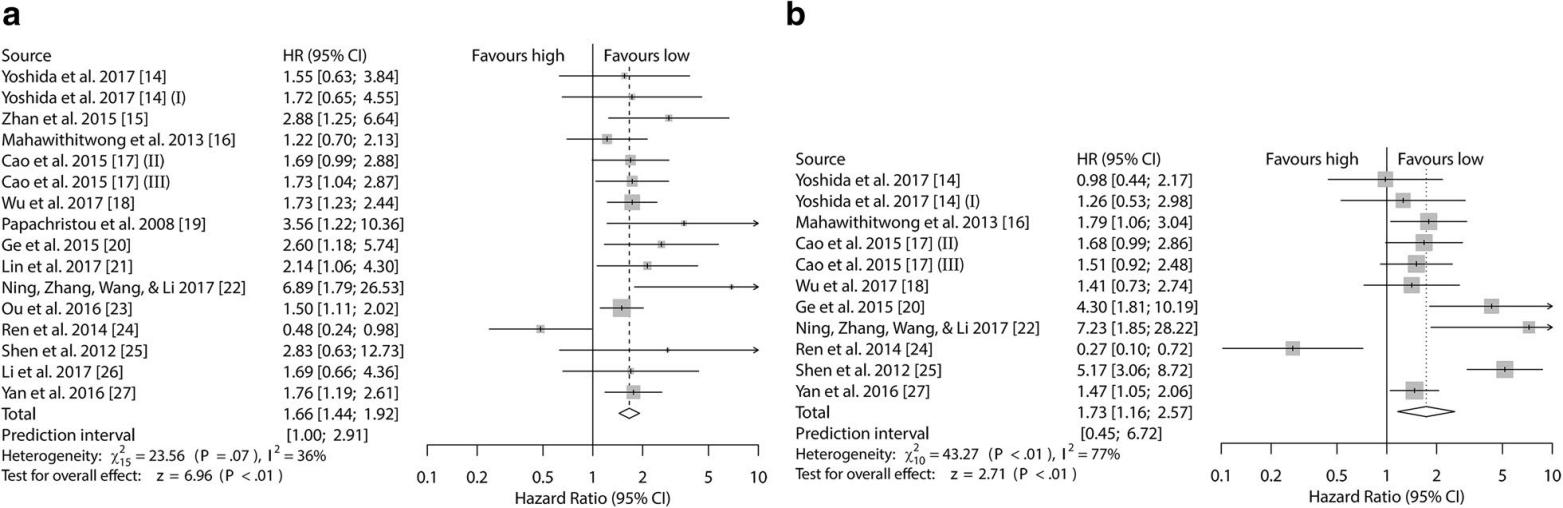
Forest plots of studies evaluating hazard ratios of high Kindlin-2 expression in solid tumors. Survival data were reported as **a** OS; **b** DFS/RFS/PFS. (I) This article (Yoshida [14]) was listed two cohort study because the sample types contain cancer tissue and startle cell. (II) and (III) This article (Cao [17]) included patients from generation dataset (II) and validation dataset (III)

For the first group, there was no significant statistical heterogeneity (I^2^ = 36.3%, *P *= 0.0729). Then, we pooled the HRs and 95% CIs by the fixed-effects model. It was indicated that high Kindlin-2 expression in cancer patients was significantly associated with a poor outcome (for OS, HR 1.66, 95% CI 1.44–1.92, *P* < 0.0001) (Fig. 2 and Table 3).

For the second group, there was obvious heterogeneity (I^2^ = 76.9%, *P* < 0.0001). Hence, the random-effects model was performed, and the correlation between high Kindlin-2 expression and poor outcomes was still statistically significant (for DFS/RFS/PFS, HR 1.73, 95% CI 1.16–2.57, *P *= 0.0067) (Fig. 2 and Table 3).

### Subgroup analysis and meta-regression analysis

**Fig. 3:**
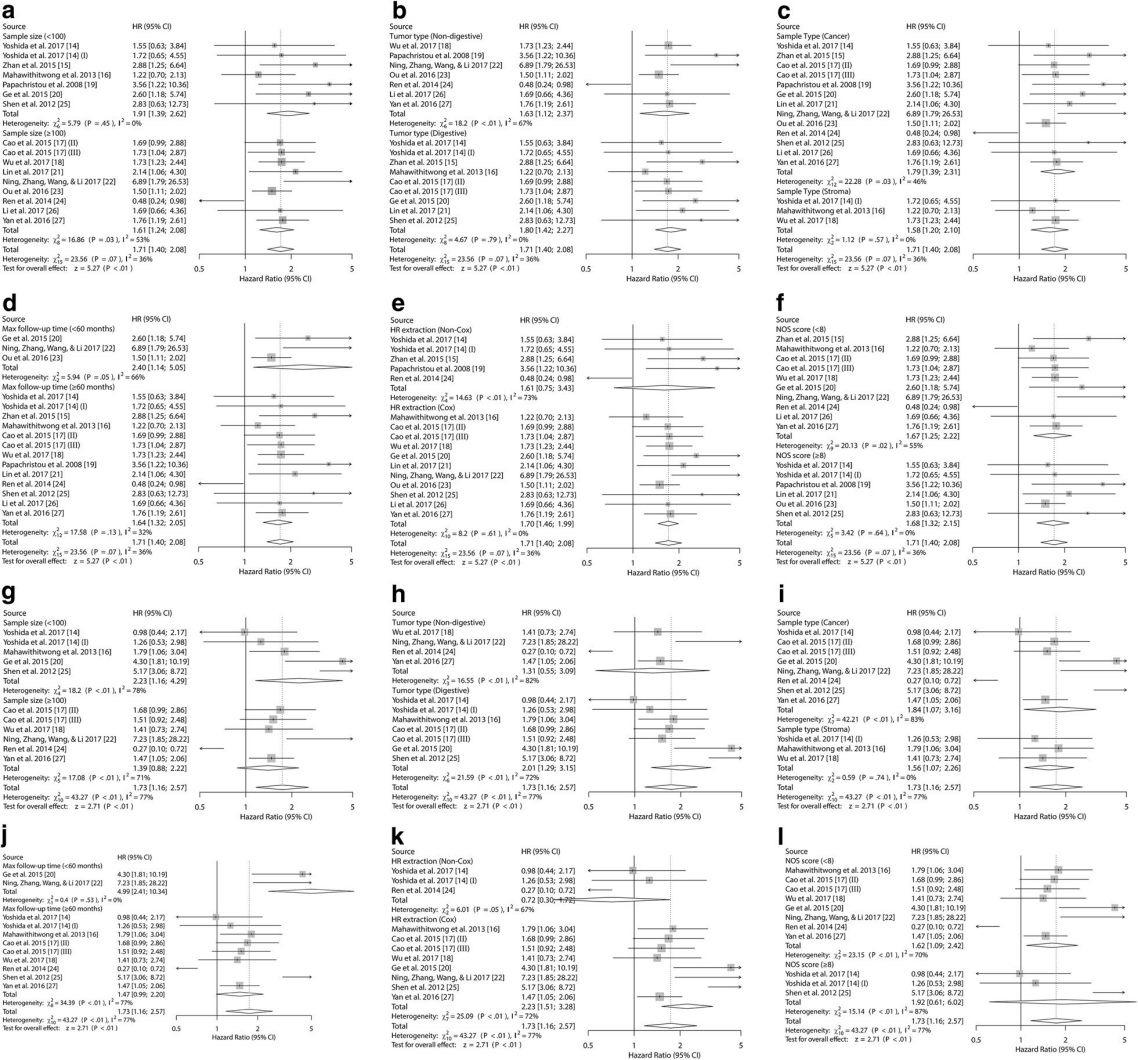
Forest plots of study subgroups according to the variables. Survival data were reported as (**a**–**f**) OS; **g**–**l** DFS/RFS/PFS. (I) This article (Yoshida [14]) was listed two cohort study because the sample types contain cancer tissue and startle cell. (II) and (III) This article (Cao [17]) included patients from generation dataset (II) and validation dataset (III)

In order to identify factors that could explain the heterogeneity of the two above groups, subgroup analysis was performed focusing on six features able to analyze: number of patients in single study (less than 100 or not), tumor type (from digestive system or not), sample type (from cancer tissue or stroma tissue), maximum follow-up time (less than 60 months or not), HR extraction (from COX model or not), NOS score (less than 8 or not) (Fig. 3 and Table 3). However, other features were not analyzed due to the deficient report or inconsistent cut-off value. Through the subgroup analysis, we found that the correlation between high expression of Kindlin-2 and OS or DFS/RFS/PFS of solid tumor patients remained significant in all features above except for the subgroup of studies with the following features: patient quantity more than 100 (for OS, HR 1.39, 95% CI 0.88–2.22, *P *= 0.1611); tumor type not from digestive system (for OS, HR 1.31, 95% CI 0.55–3.09, *P *= 0.5378); HR not extracted from COX model (for OS, HR 1.60, 95% CI 0.75–3.43, *P *= 0.2185; for DFS/RFS/PFS, HR 0.72, 95% CI 0.30–1.72, *P *= 0.4542); NOS score no less than 8 (for OS, HR 1.92, 95% CI 0.61–6.02, *P *= 0.2624) (Table 3). To explore the potential sources of heterogeneity, meta-regression analysis was performed according to the covariates including above features. The result illustrated that the above features might be not the source of heterogeneity as moderators except for maximum follow-up time (for DFS/RFS/PFS, *P *= 0.0258) and HR extraction (for DFS/RFS/PFS, *P *= 0.0085) (Table 3). Importantly, the pooled data from 11 cohorts and 1527 patients showed that Kindlin-2 could be an independent factor for prognosis of solid tumor patients (for OS, HR 1.70, 95% CI 1.46–1.98, *P *< 0.0001; for DFS/RFS/PFS, HR 2.23, 95% CI 1.51–3.28, *P *< 0.0001) (Table 3).

### Correlation between Kindlin-2 expression and survival outcomes of specific tumor types

**Fig. 4:**
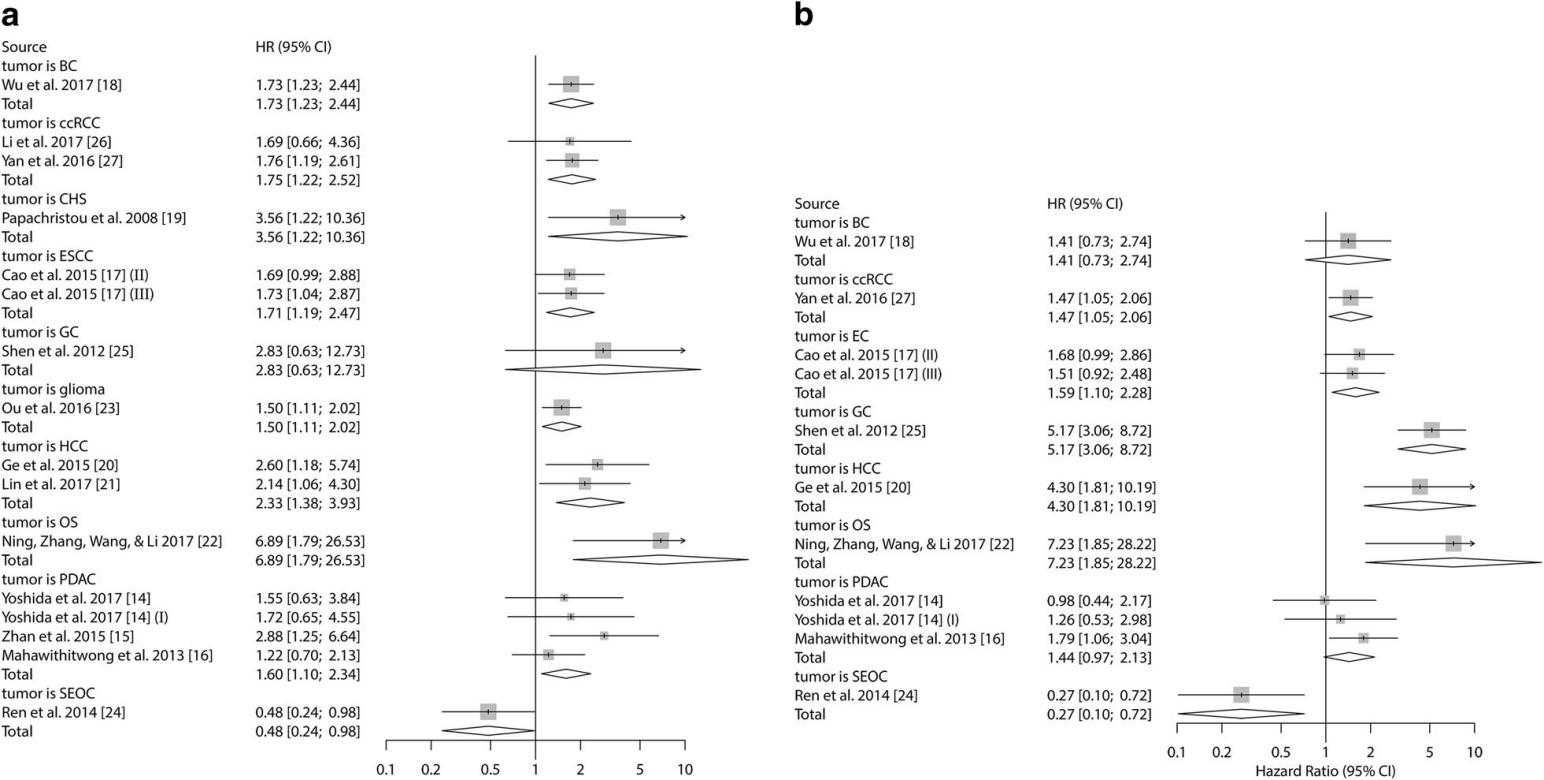
Forest plots of study groups sorted according to specific tumor types. Survival data were reported as **a** OS; **b** DFS/RFS/PFS. (I) This article (Yoshida [14]) was listed two cohort study because the sample types contain cancer tissue and startle cell. (II) and (III) This article (Cao [17]) included patients from generation dataset (II) and validation dataset (III)

The prognostic value of Kindlin-2 expression in different tumors was further investigated. We found that high expression of Kindlin-2 in PDAC patients showed an obvious correlation with poor OS (HR 1.60, 95% CI 1.10–2.34, *P* = 0.015) (Fig. 4), but showed no statistically significant association with poor DFS/RFS/PFS (HR 1.44, 95% CI 0.972–2.13, *P* = 0.069) (Fig. 4). Through meta-analysis, we also observed that high Kindlin-2 expression significantly correlated with poor OS in patients with ESCC (HR 1.71, 95% CI 1.19–2.47, *P* = 0.004), HCC (HR 2.33, 95% CI 1.38–3.93, *P *= 0.002), ccRCC (HR 1.75, 95% CI 1.22–2.52, *P *= 0.003) (Fig. 4). The pooled data also showed statistically association between high Kindlin-2 expression with poor RFS/DFS/PFS in ESCC (HR 1.59, 95% CI 1.10–2.28, *P *= 0.0129), HCC (HR 4.30, 95% CI 1.81–10.19), ccRCC (HR 1.47, 95% CI 1.05–2.06) (Fig. 4).

Consistent with their original article, the remaining HRs and their 95% CI showed that high Kindlin-2 expression had a significant relation with a worse prognosis in BC (for OS, HR 1.73, 95% CI 1.23–2.44; for DFS/RFS/PFS, HR 1.41, 95% CI 0.73–2.74), CHS (for OS, HR 3.56, 95% CI 1.22–10.36), GC (for OS, HR 2.83, 95% CI 0.63–12.73; for DFS/RFS/PFS, HR 5.17, 95% CI 3.06–8.72), glioma (for OS, HR 1.50, 95% CI 1.11–2.02), OS (for OS, HR 6.89, 95% CI 1.79–26.53; for DFS/RFS/PFS, HR 7.23, 95% CI 1.85–28.22), while it had a significant association with the better prognostic outcome of SEOC (for OS, HR 0.48, 95% CI 0.24–0.98; for DFS/RFS/PFS, HR 0.27, 95% CI 0.10–0.72) (Fig. 4).

### Publication bias assessment and sensitivity analysis

**Fig. 5:**
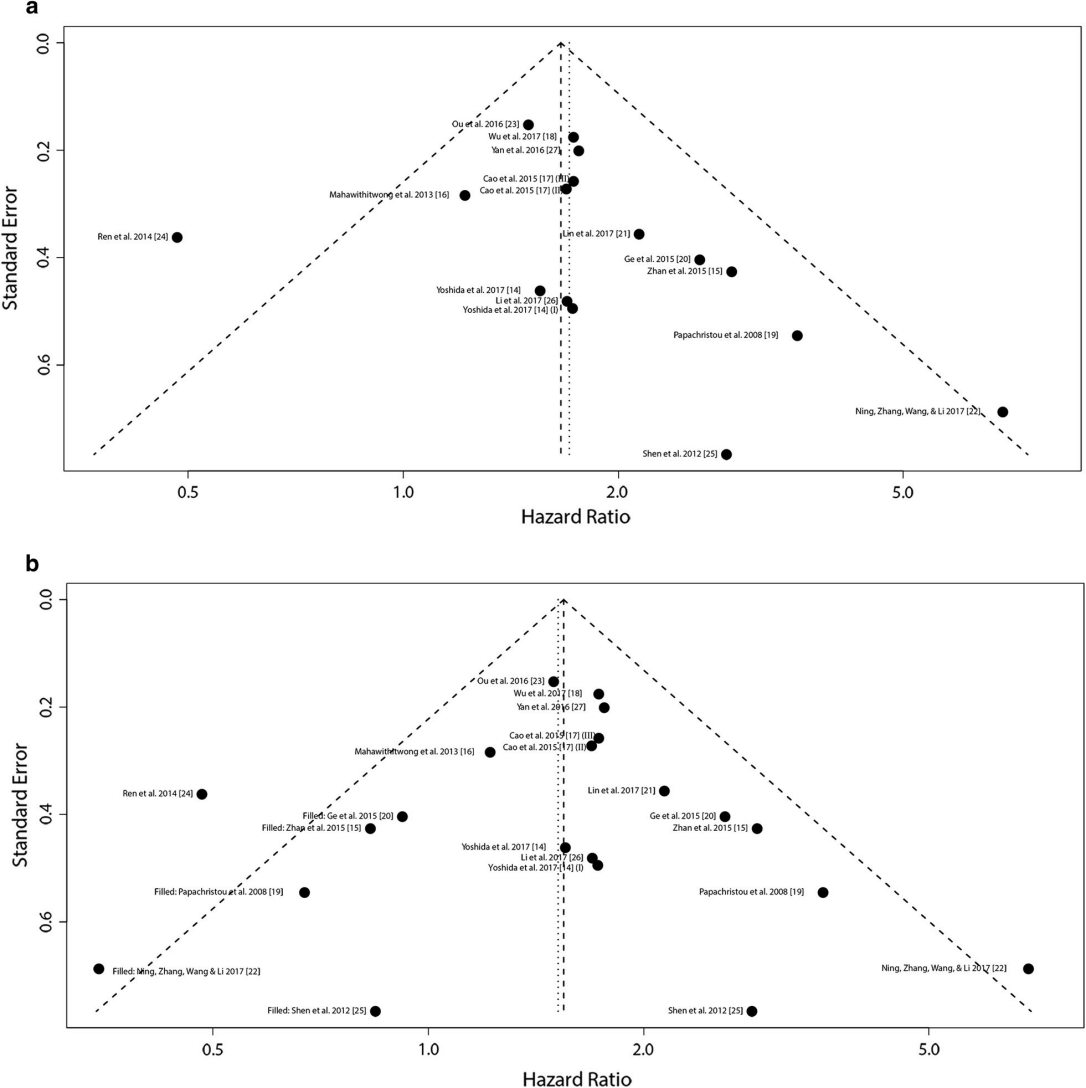
Funnel plots for assessing the publication bias. **a** Original data, **b** data rectified by the trim and filled model. (I) This article (Yoshida [14]) was listed two cohort study because the sample types contain cancer tissue and startle cell. (II) and (III) This article (Cao [17]) included patients from generation dataset (II) and validation dataset (III)

Funnel plot, Begger’s test and Egger’s test were applied to assess small-scale study effect for this meta-analysis. The plots seemed asymmetric (Fig. 5), although both Begger’s and Egger’s tests were not statistically significant (Begger’s *P *= 0.105, Egger’s *P *= 0.207). Then, we introduced trim-and-filled model to neutralize the potential bias (Fig. 5), and statistical significance of the correlation still existed (for OS, HR 1.55, 95% CI 1.35–1.77, *P *< 0.0001). Hence, no significant publication bias existed and exerted a strong impact on the pooled results in this meta-analysis.

**Fig. 6:**
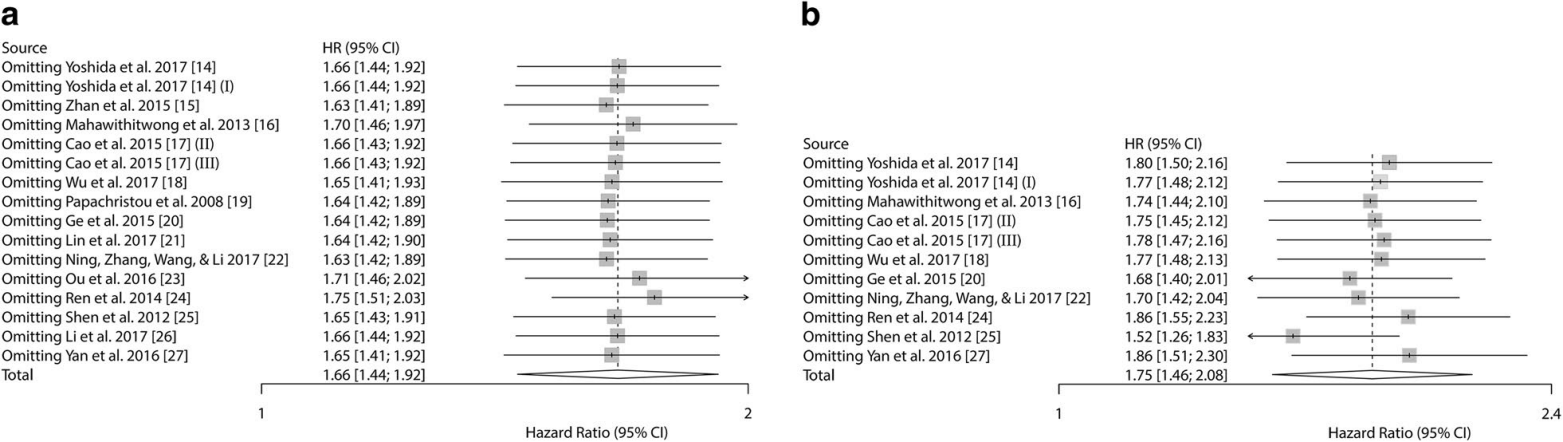
Sensitivity analysis on the prognostic value of Kindlin-2 expression in solid tumors patients. Note: Survival data were reported as **a** OS; **b** DFS/RFS/PFS. (I) This article (Yoshida [14]) was listed two cohort study because the sample types contain cancer tissue and startle cell. (II) and (III) This article (Cao [17]) included patients from generation dataset (II) and validation dataset (III)

To evaluate the effect of each study on the pooled results, we performed sensitivity analysis by omitting each single study sequentially. No study displayed an apparent influence on the overall results of OS and DFS/RFS/PFS (Fig. 6).

## Discussion

The human Kindlin-2 gene, also known as mitogen inducible gene-2 (MIG-2), was originally identified in the human diploid fibroblast cell line WI-38 by differential cDNA library screening and is located on chromosome 14q22.1 [20, 36]. Recently, increasing evidences have suggested that Kindlin-2 expression levels significantly correlate with tumor invasion, lymph node metastasis and worse survival in different cancers, such as breast cancer, bladder cancer [5]. However, Ren et al. reported that Kindlin-2 inhibited the growth and migration of colorectal cancer cells [29], and Shi et al. found that Kindlin-2 could act as a suppressor of mesenchymal cancer cell invasion [37]. Owing to limited numbers of patients and conflicting conclusion in existing studies, the association between Kindlin-2 and prognosis of cancer patients remains controversial.

To our knowledge, there is no systemic review focusing on the correlation between Kindlin-2 expression and prognosis of cancer patients. Therefore, we performed this meta-analysis for critically assessing the prognostic significance of Kindlin-2 expression and to determine whether high Kindlin-2 expression is associated with poor prognosis of cancer patients or not. Our results showed that high Kindlin-2 expression was significantly associated with poor OS of patients with various solid tumors. Meanwhile, the correlation between high Kindlin-2 expression and poor DFS/RFS/PFS was not homogenous, but still significant. Then, we performed the subgroup analysis for potential heterogeneity according to number of patients in single study, tumor type, sample type, maximum follow-up time, HR extraction, NOS score. We found that there remains an obvious relation between high Kindlin-2 expression and poor prognosis of tumor patients when concerning the above features except for the subgroups as follow: patient quantity more than 100; tumor type not from digestive system; HR not extracted from COX model; NOS score no less than 8. Given that the numbers of studies in these subgroups were limited, the correlating features may be not the source of the heterogeneity, which was consistent with the result of the following meta-regression. In the meta-regression analysis, we did found the lightly significant coefficient role in subgroup according to maximum follow-up time and HR extraction. It meant that the two potential moderators might partly account for the heterogeneity of the DFS/RFS/PFS group. Moreover, Kindlin-2 exerted a significant impact on worse prognosis of PDAC (DFS/RFS/PFS), ESCC (OS, DFS/RFS/PFS), HCC (OS), ccRCC (OS), BC (OS, DFS/RFS/PFS), CHS (OS), OSS (OS), GC (DFS/RFS/PFS) and glioma (OS), but not of PDAC (OS), GC (OS), sEOC (OS, DFS/RFS/PFS). The results revealed that Kindlin-2 expression had a varying correlation with prognostic outcomes of different tumor types. No significant publication bias existed in this meta-analysis and exerted a strong impact on the pooled result. Meanwhile, no study displayed an apparent influence on the overall results of OS and DFS/RFS/PFS. Taken together, Kindlin-2 expression could serve as a prognostic biomarker, which might help clinicians to make the best choices for cancer patients.

However, the exact mechanism behind the varying correlation of Kindlin-2 and poor prognosis has been not fully investigated. It was reported in previous studies that Kindlin-2 could be acted as an activator of integrin in the development of cancers [5]. And recent studies demonstrated that Kindlin-2 might exert a significant impact on poor prognosis by mainly modulating integrin signaling pathway and several other related signaling pathways, such as Wnt [21], TGF-β [15], EGFR [38] and miR-200b [39]. These pathways were highly related with cell proliferation, migration, invasion [23, 38, 40], vascular function [41] and epithelial-to-mesenchymal transition (EMT) program [42], which might result in the poor prognosis of patients with solid tumor. Given that integrin regulates a variety of cell functions in cancer cell, e.g. PDAC [43], inhibition of integrin signaling might be more efficient than direct inhibition of integrin. Then Kindlin-2, an essential activator of integrin, might be a promising target, which is supported by our result and a previous study reporting that several hallmarks of PDAC cell in vitro were inhabited when Kindlin-2 was stably down-regulated [15]. Previous research also concluded that embryonic dermal origins could influence the expression level of Kindlin-2 in various organs [44]. It implied that varying prognostic value of Kindlin-2 might be dependent on tumors’ embryonic dermal origins. In summary, high Kindlin-2 expression might indicate poor outcome in cancer patients and might be a promising therapeutic target for solid tumor.

Certainly, there were some limitations in our meta-analysis study. First, overall impact of Kindlin-2 expression on DFS/RFS/PFS was still inconclusive. Future study is needed to explore whether it is more accurate in predicting prognosis. Second, the number of studies for each specific tumor type there was limited. Third, the method we applied for extracting HR from KM plot was not as precise as the original study. Cut-off values of some key variables also differed among these studies. Potential heterogeneity might generate bias in the overall result. Hence, more studies with high quality are necessary for precisely illustrating the correlation between Kindlin-2 expression and prognosis of patients with various solid tumors.

## Conclusions

In conclusion, our results demonstrated that Kindlin-2 expression had a significant correlation with prognostic outcomes of patients with different solid tumors. Elevated expression level of Kindlin-2 was significantly associated with a poor prognosis in patients with PDAC (DFS/RFS/PFS), ESCC (OS, DFS/RFS/PFS), HCC (OS), ccRCC (OS), BC (OS, DFS/RFS/PFS), CHS (OS), OSS (OS), GC (DFS/RFS/PFS) and glioma (OS), but not PDAC (OS), GC (OS), sEOC (OS, DFS/RFS/PFS). More researches are warranted for accurately clarifying the association between Kindlin-2 expression and prognosis of solid cancer patients.

## Abbreviations

HR: hazard ratio
CI: confidence interval
OS: overall survival
DFS: disease-free survival
RFS: recurrence-free survival
PFS: progression-free survival
FERM: 4.1-ezrin-radixin-moesin
ILK: integrin-linked kinase
TGF-β: transforming growth factor β
EGFR: epidermal growth factor receptor
ERK: extracellular regulated protein kinases
KM: Kaplan–Meier
PDAC: pancreatic ductal adenocarcinoma
ESCC: esophageal squamous cell carcinoma
BC: bladder cancer
CHS: chondrosarcoma
HCC: hepatocellular carcinoma
OSS: osteosarcoma
sEOC: serous epithelial ovarian cancers
GC: gastric cancer
ccRCC: clear cell renal cell carcinoma
IHC: immunohistochemistry
WB: Western Blot
MIG-2: mitogen inducible gene-2
EMT: epithelial-to-mesenchymal transition
